# Developing future health professions educators’ research literacy through pedagogical journal clubs: facts and figures from five years of experience

**DOI:** 10.1186/s12909-025-07915-z

**Published:** 2025-10-02

**Authors:** Michael Ewers, Doreen Herinek, Michael Köhler

**Affiliations:** Institute of Health and Nursing Science, Charité – Universitätsmedizin Berlin, corporate member of Freie Universität Berlin and Humboldt-Universität zu Berlin, Augustenburger Platz 1, 13353 Berlin, Germany

**Keywords:** Journal clubs, Health professions education, Educational research literacy, Evidence-based education, Educators

## Abstract

**Introduction:**

Future health professions educators need to develop educational research literacy to elevate the evidence base of their educational work, and to participate confidently and substantively in academic discourse on Health Professions Education (HPE). Pedagogical Journal Clubs (JCs) could be one means to this goal, but there is little empirical evidence to support this. To address this gap, we used pedagogical JCs in a master’s program in HPE and analysed the experience gained.

**Methods:**

We conducted a self-evaluation study based on five cross-sectional online surveys from 2020 to 2024 to gather data on students’ experiences (*n* = 94) with this specific learning format. The self-developed questionnaire collected data on the respondent’s demographics, previous experiences with JCs, perceived effects of pedagogical JCs, and their potential future application. The cumulated quantitative data from these surveys was analysed descriptively, answers to a final open-ended question were clustered thematically.

**Results:**

The respondents assessed the active and critical engagement with educational research literature in the pedagogical JCs as a valuable contribution to their scientific qualification. 95% were convinced that pedagogical JCs are suitable for promoting the evidence base of their educational work. However, the master’s students were rather hesitant when asked whether they would be willing to organise and use pedagogical JCs to bridge the gap between research and practice at their future workplace. They saw numerous obstacles to such implementation in practice.

**Conclusion:**

Pedagogical JCs are a promising learning format to develop the ability to access, appraise and apply educational research results. However, further research is essential to establish a stronger evidence-based connection between pedagogical JCs and this educational research literacy. In addition, structural support is needed in institutions relevant to HPE, but so is a change in mindset among educators in order to transfer the experience gained with pedagogical JCs into later educational practice.

**Supplementary Information:**

The online version contains supplementary material available at 10.1186/s12909-025-07915-z.

## Introduction

Traditionally, all doctors, nurses, midwifes and other health professionals are expected at some point in their careers to teach students, novices, or experienced colleagues, usually in clinical environments but also in skills labs, seminar rooms or lecture halls [1–4]. Some of them might pursue a career as clinical educator, mentor, lecturer or – in general – as health professions educator and therefore seek to expand their qualifications accordingly, for example by attending one of the increasingly popular masters’ or doctoral programs in Health Professions Education (HPE) [5–8]. In these postgraduate degree programs, health professionals are confronted with educational research literature that deals with issues of teaching and learning and thus has a different thematic focus than, for example, academic literature in medicine or health sciences. This introduces them to educational theories, cultures, and methodologies that differ, at least in some respects, from those of their original health disciplines [9, 10]. In pedagogy, which is strongly influenced by the social sciences and humanities, constructivist theories of knowledge are more relevant than the positivist approaches favoured in the natural sciences, for example, and this is then reflected in the methodology and the findings developed as a result. Those who wish to become a health professions educator are expected, among other things, to familiarise themselves with the diversity of ideas, theories, and models in educational science and to develop the “ability to purposefully access, reflect, and use evidence from educational research” [11, p. 37]. This so-called ‘educational research literacy’ [12] should enable them to deal with issues and problems in educational practice or research and “to communicate competently in an academic discourse community” [13, p. 6] which might be quite new or at least less familiar to many of them [14].

Journal Clubs (JCs) are a traditional and well-recognized learning format for developing and expanding research literacy and presumably also for promoting evidence-based practice in the health professions [15–18]. JCs can be broadly defined as a group of individuals who meet regularly in person or virtually to discuss influential or interesting articles in scientific journals relevant for their professional practice. This is one of the reasons why they are widely used by health professionals of all disciplines to discuss clinical or practical problems and to keep up to date with the state of knowledge in their field of expertise [19–21]. The existing literature clearly states that JCs are an effective tool to support self-directed learning and to improve the acquisition of knowledge among health professionals [22–24]. They can also be a mechanism for developing a commitment to evidence‐based practice in health care [25]. Key factors for the success of JCs are good preparation of participants, regular meetings and, where appropriate, incentives to encourage participation, such as meetings in a less formal setting [26]. There is indication that the use of electronic tools like the internet or social media platforms can increase participation rates and the effectiveness of JCs [27–29].

Despite these advantages, there is little evidence of the sustainability of the knowledge acquired through JCs or its transfer and application to concrete problems in the participants’ professional environment [24, 30, 31]. Furthermore, there is little published research on the use of pedagogical JCs in teacher education in general [32–35] or especially in postgraduate programs for future health professions educators. Nevertheless, the few existing sources suggest that JCs with a focus on pedagogical literature could be successfully used as a teaching and learning strategy to develop educational research literacy in this context [36, 37] and thereby to enhance the ability of future educators to access, appraise and apply evidence from educational literature. In this case, pedagogical JCs could eventually also be a means of bridging the gap between educational research and practice in HPE [38].

This assumption was the starting point for using pedagogical JCs in a master’s program in Health Professions Education at a large medical faculty in Germany. Although students on this programme may already have some prior experience of critically evaluating research literature due to their bachelor’s degree or other previously acquired academic qualifications, they were introduced to relevant educational research literature for the first time in the pedagogical JCs of the master’s programme. In this article, we report on facts and figures from five years of experience with this learning format in this specific context. With our research we wanted to find out whether future health professions educators consider pedagogical JCs to be beneficial for developing their own educational research literacy and research skills, whether they find this kind of JCs a helpful tool to make their future teaching and mentoring practice in HPE more evidence-based, and what they think about conducting and using pedagogical JCs together with their colleagues at their future workplaces. By answering these research questions, we aim to make an empirically based contribution to the promotion of educational research competence and evidence-based education in the postgraduate training of health professions educators.

## Materials and methods

### Study design and context

**Fig. 1:**
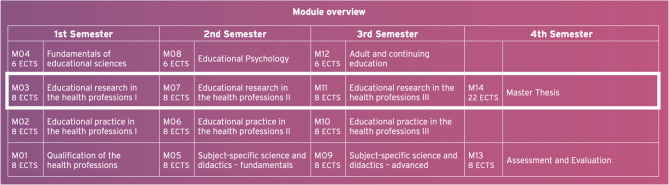
Module overview of the master’s program in HPE at Institute of Health and Nursing Scienceat Charitéé – Universitäätsmedizin Berlin

We conducted a self-evaluation study based on five cross-sectional surveys from the years 2020–2024 to gather students’ experience with pedagogical JCs which were offered once a year in one of the modules of the four-semester postgraduate degree program mentioned above. A total of four compulsory modules on “empirical educational research” are offered in this full-time program with 120 Credits following the European Credit Transfer System (ECTS), one per semester. The first module (M03) serves to refresh and supplement existing knowledge and skills in various research methodologies and methods (quantitative, qualitative, mixed-methods, literature reviews). The second module (M07) introduces a variety of empirical research fields on HPE – including instructional research, teacher education research, vocational training research and higher education research. This module consists of a lecture and a seminar, which is designed as a reading course and includes the self-organised pedagogical JCs. The third module (M11) is used for the supervised development of an exposé on selected topics of empirical educational research, while the fourth module (M14) is geared towards the independent preparation of the research master’s thesis. Overall, the four modules aim to develop students’ skills in finding, understanding, appraising and using educational research in HPE, i.e. to build their so-called educational research literacy (see Fig. 1).

As an admission requirement for the HPE masters’ program, the students must provide a license to practice in a nationally regulated health profession and a first academic degree with at least 180 ECTS. Most students have a background in either nursing, midwifery, or allied health, some of them also have several years of clinical practice in various health care settings. In Germany, the academization of these professions is still at a very low level compared to other European countries, which is why most of them acquire their licence via vocational training in traditional secondary hospital-based schools. These vocational training programmes are classified at level 4 of the 8-level European Qualifications Framework (EQF) [39]. They primarily impart factual and theoretical knowledge as well as practical skills for working in contexts that are generally predictable but may be subject to certain changes. Scientific skills and research methods are usually not part of the curriculum. First academic degree programmes, on the other hand, are classified at level 6 and impart advanced knowledge combined with the ability to critically understand theories and principles, this includes an introduction to scientific skills and research methods. They are designed to develop broad, transferable skills to cope with complex professional activities in unpredictable work contexts. A master’s degree is classified as level 7, a doctorate as level 8 of the EQF [39].

The graduates of the HPE masters’ program usually aim to work as a teacher in one of the traditional vocational schools. In some professions, however, legal developments have already been initiated in the form of a partial (for nurses) or full transfer (for midwives) of preregistration education to the tertiary level of the education system. These options were opened in the winter semester of 2020/2021 following the enactment of the new professional laws. Because of this, graduates of the master’s program in HPE might also work as faculty member in some of the new degree programs or as scholars at universities or universities of applied sciences (UAS). Finally, there is the opportunity to take on different educator roles in the continuing professional development of health professionals – for example at training facilities or skills labs of large hospitals. For all these roles and responsibilities, future health professions educators not only need access to current pedagogical knowledge and the ability to critically evaluate the scientific educational literature. They should also be able to provide a solid evidence base for educational practice and, where appropriate, contribute to the discipline’s body of knowledge through their own educational research [7, 40].

### Intervention

To develop their educational research literacy, students of the master’s program in HPE were asked to prepare and conduct pedagogical JCs as part of the seminars included in module M07. In these self-organized JCs, four research articles with different educational topics were presented, evaluated using critical appraisal tools and discussed regarding their relevance for everyday educational practice in HPE. The studies to be critically evaluated were chosen by the module coordinators (ME, DH, MK) to achieve a uniform level of academic performance. Otherwise, the organisation and implementation of the pedagogical JCs followed the usual standards for this learning format [41]. Accordingly, each cohort took part in a total of four journal club meetings during a semester. The pedagogical JCs were prepared by different student groups on different topics with each meeting lasting 90–120 min.

### Data collection and questionnaire

At the end of the semester, all students who participated in module M07 were asked to take part in an online survey to evaluate their experiences with the pedagogical JCs. The students could only access the questionnaire after they had read the ethical and data protection information and given their full consent to participate voluntarily in the study. The self-developed questionnaire included scales for students to report their past experiences with JCs in other contexts, to rate their experiences of the pedagogical JCs in the master’s program in HPE, and to assess their usefulness for their educational research skills and future educational practice in HPE (see supplementary file). For example, the students were asked whether the pedagogical JCs were instructive from a technical, methodological, or pedagogical point of view or how likely it was that participation has increased their (educational) research skills and literacy. Students were also asked whether they would use pedagogical JCs in their future workplaces in collaboration with other health professions educators to improve the evidence base of their teaching or mentoring, or what would be the most likely reasons for not implementing and using them regularly. At the end of the questionnaire, they could state in an open question what else was important to them regarding the pedagogical JCs.

### Data analyses

The cumulated data from five years was cleansed and processed and then analysed descriptively with frequencies, mean values and standard deviations being specified. The free text data was subject to inductive thematic clustering [42]. The first round of clustering themes was carried out independently by two authors (MK/DH), followed by a second round in which all authors of this article discussed the clusters and agreed on the relevant themes. Findings were summarised in a condensed form.

## Results

### Sample characteristics

A total of 94 master students participated in the five surveys, 78 of whom identified themselves as female (85.7%) and 13 as male (14.3%). Slightly more than two-thirds stated they were between 25 and 34 years old at the time of the survey. The largest group was made up of nurses with a total of 50 participants, while midwives were the smallest with 8 students. More than a third of the respondents were in allied health professions (occupational therapy, physiotherapy, speech and language therapy). Approximately two-thirds (63,8%) of the students obtained their professional license through a traditional vocational training in the secondary education sector. The remaining third (36,2%) got their license through one of the in Germany relatively new undergraduate degree programs offered from UAS or universities. All characteristics of the sample are outlined in Table 1.

**Table 1:** Sample characteristics

Characteristics		Frequencies	n
		%	
Sample		100%	94
Professional background	Allied health profession	35.5 %	33
	Midwife	8.6 %	8
	Nurse	53.8 %	50
	Other	2.2 %	2
Professional licensure via	Traditional vocational training (secondary education)	63.8 %	60
	Undergraduate degree program (tertiary education)	36.2 %	34
Sex	Female	85.7 %	78
	Male	14.3 %	13
	Diverse	0.0 %	0
	Not specified	0.0 %	0
Age (years)	≤ 24	14.3 %	13
	25-34	69.2 %	63
	35-44	13.2 %	12
	45-54	3.3 %	3

Nearly half of the participants (*n* = 46) had already experience in some sort of teaching (in different settings). Around a quarter of respondents (*n* = 25) had previous experience with JCs. Most of these students (*n* = 22) acquired this experience during their first-degree program, only a few students encountered JCs via social forums on the internet (*n* = 3), working groups in professional associations (*n* = 2) or in specialist journalistic work (*n* = 1). None of the respondents stated that they had previous experience in the use of JCs during their professional training or clinical work. If the respondents had already gained experience with JCs before the master’s program in HPE, then it was primarily either with a clinical-practical (37.5%), research-methodological (37.5%) or content-related (25.0%) focus.

### Developing educational research literacy

Students were asked how likely it was that participating in pedagogical JCs had improved their educational research literacy. Participants rated the likelihood on a scale of 0 (very unlikely) to 10 (very likely) with an average of M = 7.1 (SD = 2.0), thus as rather likely. The students emphasized to benefit particularly in terms of research methodology, as noted by 73 students (77.7%). Regarding its professional aspect, 15 participants (16.0%) found it instructive, while only 6 respondents (6.4%) reported pedagogical benefits.

**Fig. 2:**
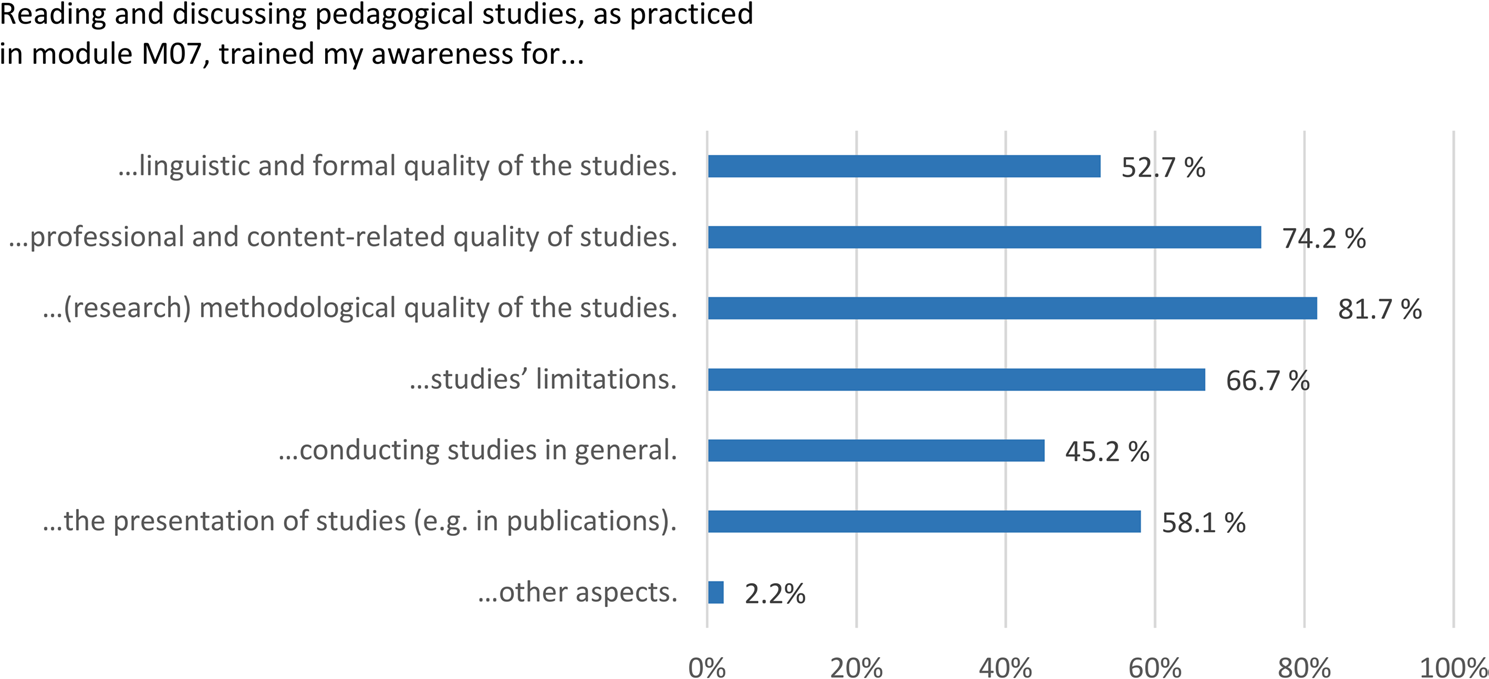
Aspects of research literacy for which awareness was raised through participation in pedagogical JCs (multiple answers possible)

A differentiated picture of the benefits of pedagogical JCs can be seen in Fig. 2. In this multiple selection, the students predominantly found that engagement in pedagogical JCs notably sharpened their awareness for the methodological quality of educational studies (82%), followed by professional and content-related quality of research (74%) and the awareness for studies’ limitations (67%). Slightly fewer but still more than half of the respondents stated that the pedagogical JCs had trained their attention regarding the presentation (58%) as well as the linguistic and formal quality of scientific studies (53%). The least frequently selected aspect was attention to conducting scientific studies in general (45%).

### Promoting evidence-based practice in HPE

**Fig. 3:**
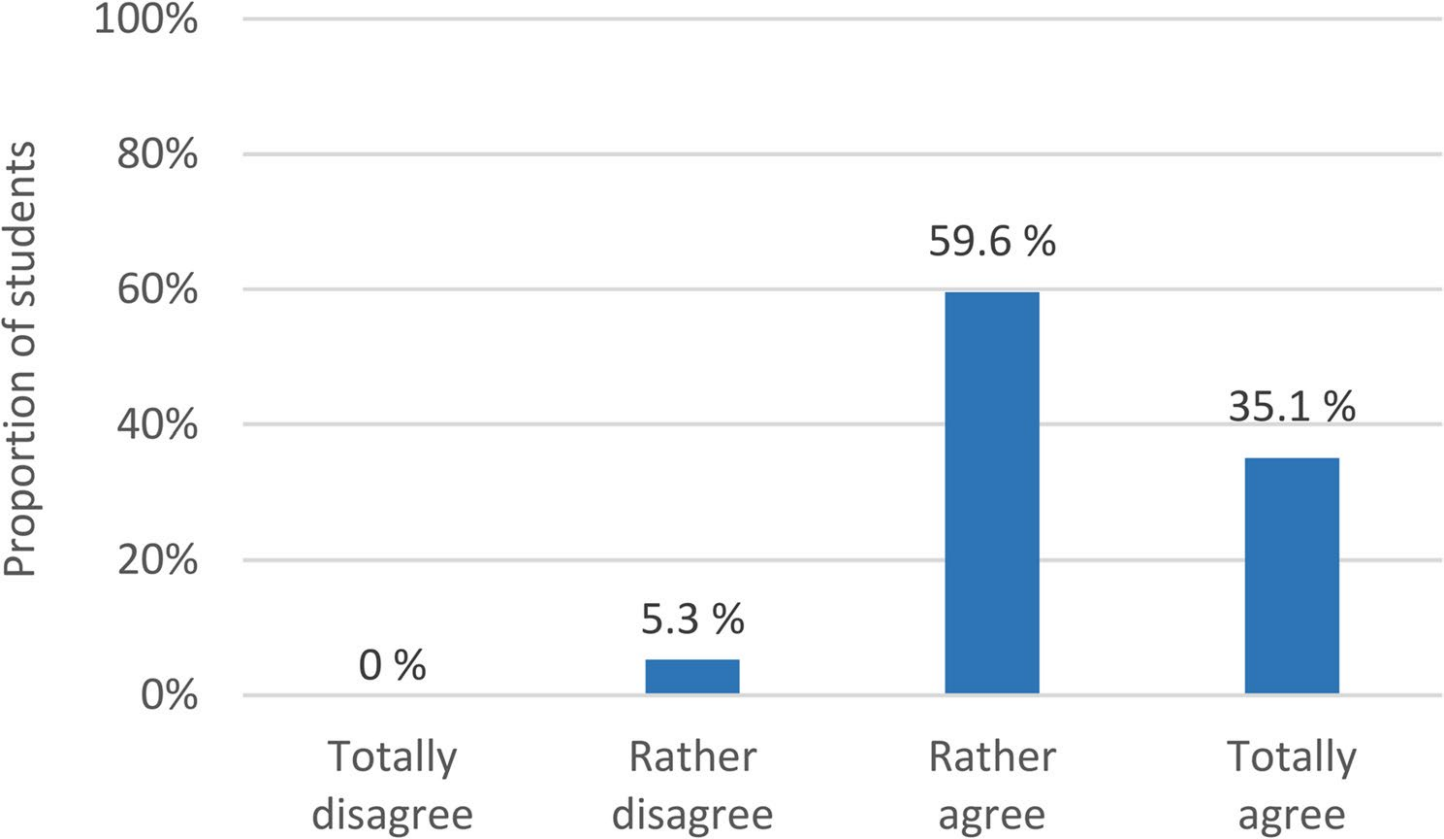
Assessment of whether pedagogical JCs can promote evidence-based educational practice in HPE

Students were asked how likely it is that pedagogical JCs contribute to improve education and training in the healthcare professions based on an evidence-based approach. Their average rating on the scale from 0 (very unlikely) to 10 (very likely) is 6.9 (SD = 2.4). 35% of students totally agreed and 60% rather agreed that the pedagogical JCs are an important part of health professions educators’ work and that they can promote evidence-based HPE. A small proportion (5%) rather disagreed with the statement, but no one completely disagreed (see Fig. 3).

### Likelihood to use pedagogical JCs in educational practice

**Fig. 4:**
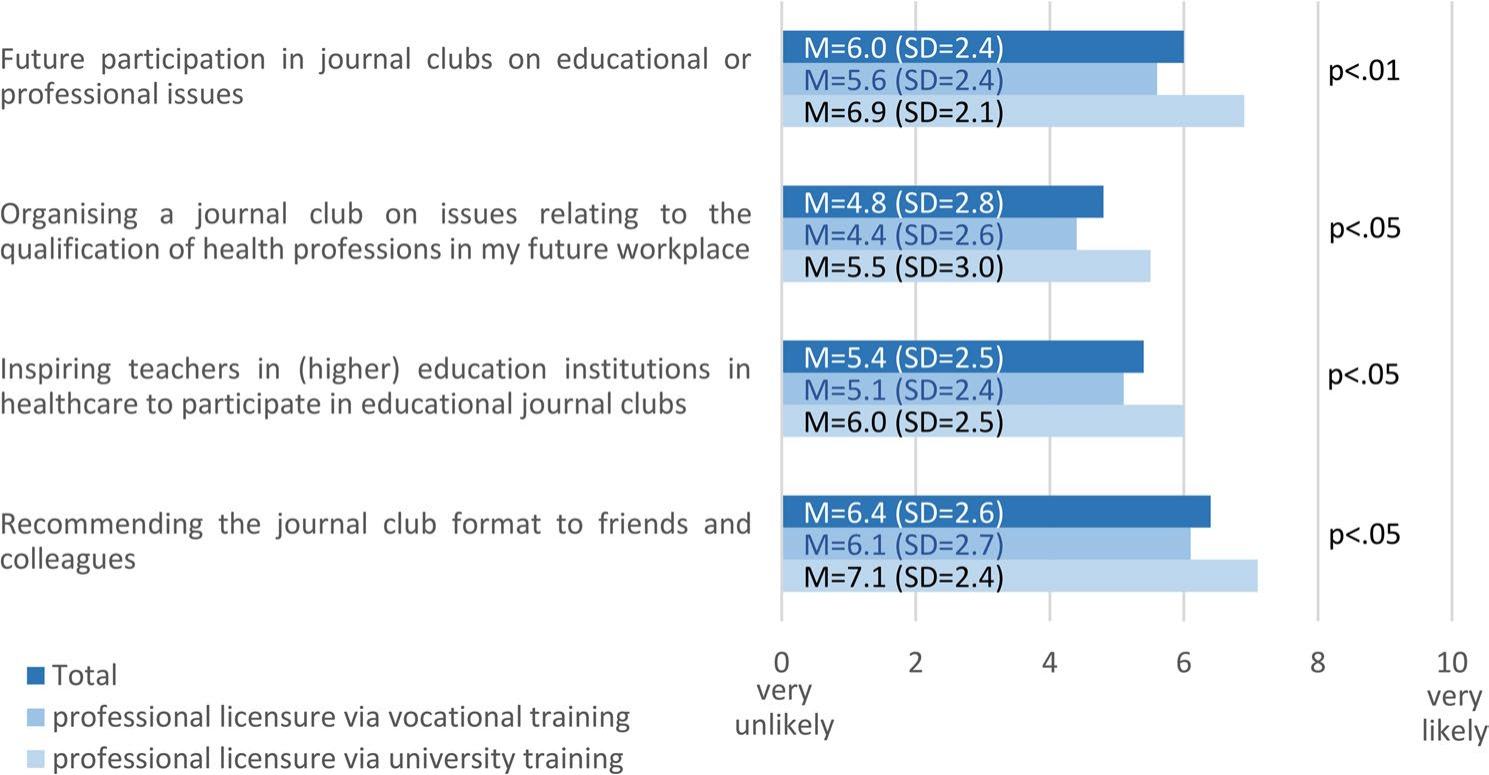
Self-estimated likelihood to use pedagogical JCs in educational practice

When asked whether they would participate in pedagogical JCs in the future, the participants responded on average with M = 6.0 (SD = 2,4) on the scale from 0 (very unlikely) to 10 (very likely). Even more of them considered it likely that they would recommend pedagogical JCs to friends and colleagues (M = 6.4; SD = 2.6). However, they thought it less likely that they would organize and conduct pedagogical JCs in their future workplace (M = 4.8; SD = 2.8). They were also rather unsure, if they could inspire other health professions educators in their future workplaces to participate in pedagogical JCs (M = 5.4; SD = 2.5). The results of this question are summarised in Fig. 4. This figure also shows differences between students who obtained their professional licence through vocational training and those who completed a course of study to do so. The latter group on average considered it somewhat more likely that they would use pedagogical JCs in their educational practice in the future.

**Fig. 5:**
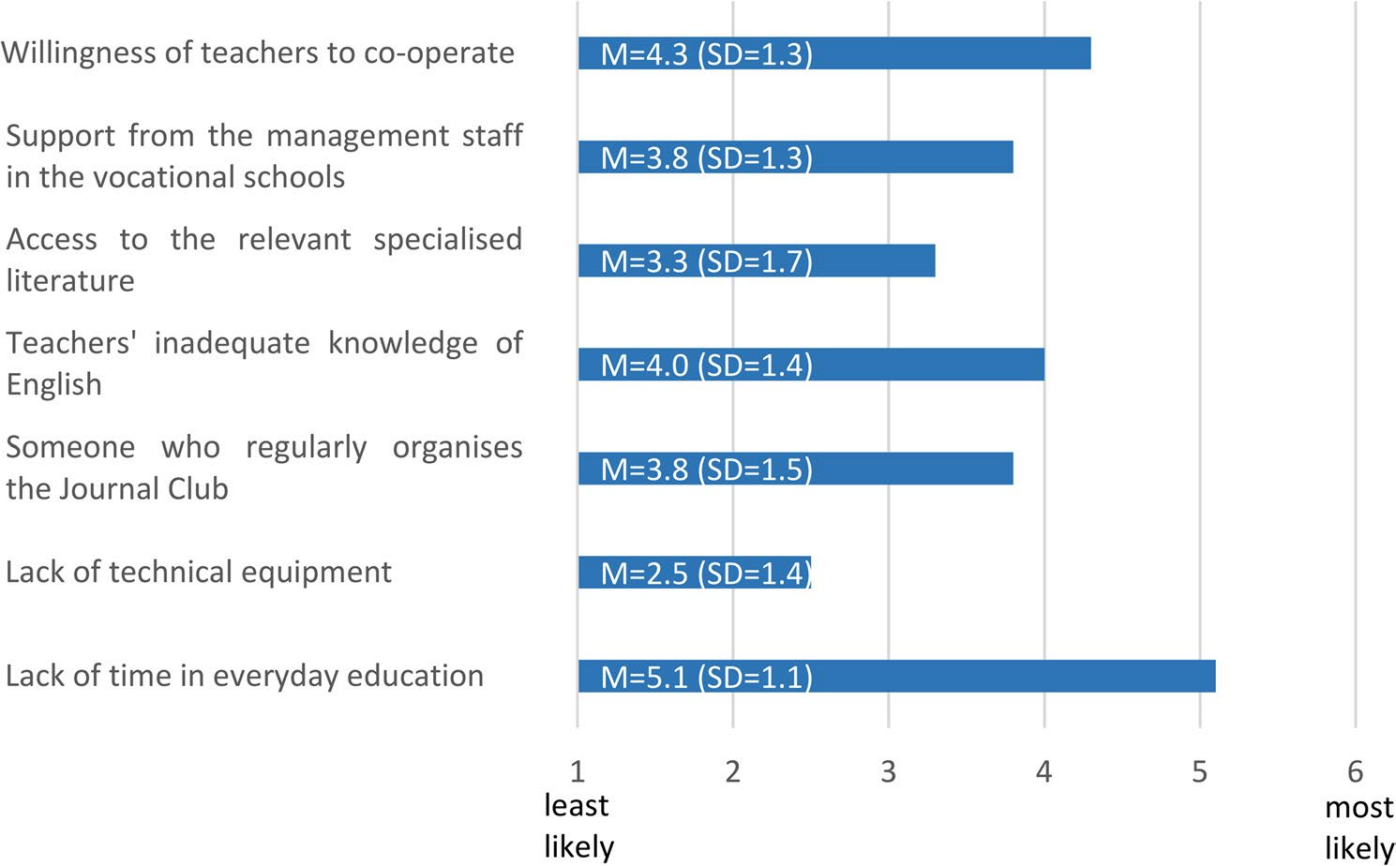
Possible obstacles to the establishment of pedagogical JCs in everyday practice in HPE

Finally, participants were asked to rate various factors that could affect the likelihood of implementing and using pedagogical JCs in their future educational work in HPE. On the scale from 1 (least likely) to 6 (most likely), participants identified the lack of time as the most likely barrier to organizing and conducting JCs with the teacher colleagues (M = 5.1; SD = 1.1). In addition, lack of willingness to cooperate (M = 4.3; SD = 1.3), inadequate English language skills (M = 4.0; SD = 1.4), insufficient support from the management staff in the vocational schools (M = 3.8; SD = 1.3) as well as to find someone who regularly organises the pedagogical JCs (M = 3.8; SD = 1.5) were cited as possible obstacles. Insufficient access to relevant educational literature (M = 3.3; SD = 1.7) and a lack of technical equipment (M = 2.5; SD = 1.4) were considered by students to be less likely obstacles to using pedagogical JCs (see Fig. 5).

### Other relevant aspects regarding the use of pedagogical JCs in HPE

A total of 25 participants from the five surveys took the opportunity to answer a final open question about what else was important to them in relation to pedagogical JCs. The answers were categorised into three thematic clusters: Benefits, Challenges, and Barriers. Table 2 shows that the three topic clusters were each specified by several subtopics. In addition, sample quotations are documented to illustrate the experiences and considerations of the respondents.

**Table 2:** Thematic clusters, sub-topics and sample quotes from final open question

Cluster	Sub-topics	Sample quote
Benefits	• Skills acquisition (content-related and methodological) • Opportunity for professional exchange • Trains the handling and appraisal of studies • Impetus for change (evidence-based education) • Having fun during the realisation	*“[…] that this dynamic exchange provides very low-threshold access to complex scientific issues and that the rather informal exchange promotes individual ideas and approaches. Furthermore*,* the method could initiate targeted developments/changes in a group of teachers.”*
Challenges	• Lack of Time • High workload • Lack of personal resources (willingness to take part; different levels of competence; voluntariness and moderation)	*“As far as schools are concerned*,* teachers are very busy and don’t know how to manage the workload. This will be a challenge*,* especially for those who want to organise a journal club in their school*,* to convince their overworked colleagues to reprioritise and then take part in a critical reading group despite their heavy workload.”*
Barriers	• Transfer • Lack of practical relevance • Experience-based action	*“However*,* it’s also the case that the studies […] can’t always be transferred to my small school. Different teacher training*,* different subject*,* different country*,* etc. But you mustn’t forget one thing: the experience factor*,* which in my opinion outshines even the best study. If someone has been a teacher for 20 years or more*,* they simply know from their wealth of experience how to give good lessons*,* what works and what doesn’t work - leaving aside burnout and resignation*,* which can occasionally be found in older teachers.”*

## Discussion

The aim of this study was to make an empirically based contribution to the promotion of educational research literacy and evidence-based education in the postgraduate training of future health profession educators. By gathering and evaluating experiences with pedagogical JCs in a German master’s program in HPE, we wanted to find out whether this special learning format could be a means of promoting the development of research skills and educational research literacy in future health professions educators while also bridging the gap between educational research and teaching practice and thereby promoting evidence-based HPE.

Our observations from five years of experience with pedagogical JCs confirmed that this specific learning format is a valuable tool for developing future health professions educators’ research literacy. The main benefit of this learner-centred format was perceived in the methodological field, which is consistent with earlier research on this topic [22, 41]. Preparing for and participating in self-organised educational JCs can significantly influence awareness of the methodological quality of educational studies. The critical appraisal of educational research literature provides the opportunity to learn about different study designs and their benefits and limitations, the presentation of research results and the discussion of findings. Pedagogical JCs offer opportunities for the participants for active engagement and self-directed learning, as well as for higher-level knowledge transfer through intensive debates [35]. Based on our observation, participating in the JCs has changed the way students in the master’s programme in HPE engage with educational research. Students who had previously only been involved in clinical research were given the opportunity in the JCs to familiarise themselves with educational research in the field of HPE and its topics, theoretical and methodological approaches, and specific challenges. Even though this advantage was considered less important by the respondents in our surveys, it is still noteworthy and should encourage a more intensive use of pedagogical JCs in the training of future health professions educators [33, 36].

Participation in the pedagogical JCs was associated with high expectations in terms of a stronger evidence base for educational practice in HPE. The intensive study of educational literature on the education and training of health professionals was considered helpful by the prospective educators to select the most suitable teaching and instruction methods for a specific learning objective, situation, group of learners or context. Most of the respondents felt supported in their informed decision-making on critical issues in HPE. Finally, critically reading international literature on selected topics and educational research fields – such as instructional research, comparative educational research or media research – offered the participants suggestions for their own research work in HPE (for example, regarding their later master’s thesis). This positive attitude towards educational research, its results and their implementation in educational practice, as shown by the prospective health professions educators in our research, is in line with the results of previous studies on this topic [35, 37]. Pedagogical JCs can support the development of “knowledge communities” [38] that are particularly important for the professional development of future educators in HPE. They can be a place to keep one-self up to date with current research results, for reflective practice and finally for quality development and innovation in this specific field of educational work.

At the same time, our research provided evidence that transferring the advantages of JCs into future working environments and thus into everyday practice poses a particular challenge for future educators, which could prove to be a bottleneck when it comes to closing the gap between educational research and practice in HPE [1, 3, 38]. The Implementation of pedagogical JCs in the various teaching and learning environments was viewed with a certain scepticism by the future health professions educators, particularly when corresponding expectations are placed on them. It is noteworthy, however, that in this case, factors such as a lack of language skills or access to literature were not considered to be decisive. Rather, motivating their overworked colleagues in educational practice to participate in pedagogical JCs and “engaging the disengaged” [34] was seen by the respondents as main obstacle. On the one hand, this finding emphasises the need to strengthen leadership and change agent functions in future health professions educators [5–7].

In this context, it is important to mention that students who have obtained their professional licence through academic degree programs consider it more likely than their colleagues who have completed vocational training to organise a pedagogical JC at their future workplace and to motivate other educators to participate in it. This could indicate that undergraduate degree programs for health professionals are better at preparing students for leadership and change agent roles than the traditional vocational training. In fact, such roles and tasks are an integral part of an education at level 6 of the EQF. This form of education aims at the knowledge- and evidence-based further development of practice, even under unpredictable conditions, and at the same time at the ability and willingness to “take responsibility for the professional development of individuals and groups” [39] (p. 19). People with a master’s degree (EQF level 7) should also be able to “take responsibility for the further development of specialist knowledge and professional practice and/or for reviewing the strategic performance of teams” [39] (p. 19).

On the other hand, the findings of this study show that long-term changes towards evidence-based educational practices cannot be expected of junior educators alone. Rather, structural support measures in vocational schools, faculties and practical teaching settings, as well as a targeted change in the mindset of established health professions educators, would be required. As already suggested in the literature [12, 31, 33], pedagogical JCs should receive institutional support and be included in the routine scholarly activities of all institutions and settings who are responsible for the education and training of the next generation of health professionals. Such measures could offer long-term sustainability of pedagogical JCs and at the same time relieve the burden on the prospective educators and offer them the opportunity to get actively involved in knowledge and practice communities in HPE.

### Limitations

Besides the benefits of this self-evaluation study and the valuable insights into the development of future health professions educators’ research literacy through pedagogical JCs this research provided, there are also some limitations to consider. The study makes no claim to causal associations, as it involves five individual cross-sectional surveys whose data were analysed together. Due to the lack of a longitudinal design, no statements could be made about changes over time, for example in research literacy of the participants. And no statements can be made about differences in the annual conditions of the cohorts. The self-developed instrument was neither intended nor designed to produce reliable data on causal relationships between participation in the pedagogical JCs and actual educational research competence, nor to measure an increase in competence on the part of the students. Rather, it served to record the students’ individual experiences with this teaching and learning format and to gather their perceptions of its potential use in their future teaching practice. The results are not generalizable because the sample size was relatively small and conveniently selected. They may also have limited transferability because of the composition of the students and the specific features of the programme (e.g., the consecutive module structure of the master’s program in HPE). Since the students could only speculate about if and how they would implement pedagogical JCs in their future work environments, the statements on this should also be treated with some caution. Specific investigations into possible practical implementation were beyond the scope of this study. However, further research could be carried out to investigate how pedagogical JCs can be sustainably implemented in educational institutions that are relevant for HPE.

## Conclusion

This study provided valuable contributions to the relatively limited research on the use of pedagogical JCs as a means to develop educational research literacy, especially in programs for future health professions educators. Further methodologically sound studies are needed to establish a causal link between participation in a pedagogical JC and the ability of health professions educators to access, appraise and apply evidence from educational literature. However, there are already growing indications that pedagogical JCs are “a potentially powerful pedagogy” [26, p. 240] and that prospective educators in the health professions can benefit from this specific learning format. It might make it easier for them to familiarise themselves with the diversity of ideas, theories and models in educational science, discuss subject-specific content with other educators at an academic level, critically evaluate the results of educational research and apply them in their teaching practice. However, transferring the experience gained with pedagogical JCs into later educational practice proves to be the most difficult barrier for the future health professions educators to overcome. To counter this, structural support measures are needed in the relevant institutions, but so is a change in mindset among educators and leaders already working in HPE. Integrating pedagogical JCs into vocational schools or faculties offering programs for the education and training of health professionals could be a means to support the adoption of evidence-based educational practices in HPE, to stimulate change in teaching and learning and to build up communities of knowledge and practice in this field. 

## Supplementary Information

Supplementary Material 1.

## Data Availability

The datasets used and/or analysed during the current study are available from the corresponding author on reasonable request.

## Abbreviations

JC: Journal Clubs
HPE: Health Professions Education
ECTS: European Credit Transfer System
UAS: Universities of Applied Sciences
SD: Standard Deviation
M: Median
p: p–value
