# Supplementary material for: Developing future health professions educators’ research literacy through pedagogical journal clubs: facts and figures from five years of experience
Source: BMC Med Educ. 2025 Oct 2;25:1278. doi: 10.1186/s12909-025-07915-z (PMC12490084; doi:10.1186/s12909-025-07915-z)
Supplement: Supplementary file 1 — Supplementary Material 1. [file 12909_2025_7915_MOESM1_ESM.docx]

**Survey on Pedagogical Journal Clubs in Module M07 – Empirical Educational Research**

**of the Master’s Degree Program Health Professions Education**

**at Charité – Universitätsmedizin Berlin***

Dear students, with this survey we would like to record and evaluate your experiences with the Pedagogical Journal Clubs in module M07 of the Master Health Professions Education. We want to use the results of the survey for the further development of the module, but possibly also to prepare a scientific article about the development of your research literacy. We need your support for this.

The data collected here will be used exclusively for scientific purposes and processed in anonymised form. It will NOT be possible to identify you personally or to attribute individual statements directly to you later. Participation in the survey is voluntary, and you can stop answering the questions at any time without giving reasons. Answering all the questions will take about 10-15 minutes. We thank you for your willingness to collaborate in this educational research project.

1. **Some general questions about yourself**

In this section of the questionnaire, we ask you some general questions about yourself to help us to better categorise and analyse the information from this survey later.

1. I have a professional qualification or a professional licence in one of the following healthcare professions... (please tick as appropriate)

- midwifery
- nursing (elderly)
- Nursing (general)
- nursing (paediatric)
- occupational therapy
- physiotherapy
- speech & language therapy
- miscellaneous

1. I have my professional licence... (please tick as appropriate)

- acquired as part of a vocational training programme
- acquired as part of a degree programme

1. Sex (please tick as appropriate)

- female
- male
- miscellaneous
- not specified

1. Age (please tick as appropriate)

- up to 24 years
- 25 - 34 years
- 35 - 44 years
- 45 - 54 years
- 55 years and older
- Not specified

1. I already have experience as an educator (please tick as appropriate)

- no
- yes

1. I have experience as an educator... (multiple answers possible)

- collected in patient training / health education
- in health professions education
- during my studies at the MHPE
- miscellaneous

1. I was introduced to the Educational Journal Clubs in the following term... (please tick as appropriate)

- summer term 2020
- summer term 2021
- summer term 2022
- summer term 2023
- summer term 2024

**B. Questions about Journal Clubs in general**

This section of the questionnaire is about your experiences with Journal Clubs in general and with the Education Journal Clubs as part of Module M07 in the master’s programme Health Professions Education in particular. Please note: This is not about whether these Journal Clubs were held in person or online.

1. I already had experience with Journal Clubs before my seminar in M07 (please tick as appropriate)

- No
- Yes

1. My experiences with Journal Clubs outside the seminar in M07 come from... (multiple answers possible)

- my professional training
- my first-degree programme (Bachelor or similar)
- my professional / clinical practice
- working groups in professional organisations
- social forums on the internet (online JC)
- miscellaneous

1. In the journal clubs I have been involved in outside of module M07, the focus has been on... (please tick as appropriate)

- clinical-practical questions
- deals with scientific questions
- research methodological questions
- pedagogical issues (training/further training)
- Miscellaneous

1. Reading and discussing studies in the Journal Club as part of module M07 was... (please tick as appropriate)

- particularly instructive from a technical point of view
- particularly instructive from a methodological point of view
- particularly instructive from a pedagogical point of view
- in no way instructive

1. Reading and discussing pedagogical studies, as practised in module M07, has trained my awareness for... (multiple answers possible)

- the linguistic and formal quality of studies
- the professional and content-related quality of studies
- the (research) methodological quality of studies
- the limitations of the respective studies
- the conduct of scientific studies in general
- the presentation of scientific studies (e.g., in publications)
- miscellaneous

1. How likely is it that participation in the Journal Club has increased my research literacy? Research literacy here means the ability to search for, find, understand, evaluate and use research literature on selected topics for your own work. (please position your rating on the scale)

0 1 2 3 4 5 6 7 8 9 10

Extremely unlikely Extremely likely

1. How likely is it that Journal Clubs on pedagogical topics can contribute to improving education and training practice in the healthcare professions? (please position your rating on the scale)

0 1 2 3 4 5 6 7 8 9 10

Extremely unlikely Extremely likely

1. How likely is it that I will recommend the Journal Club format to friends or colleagues? (please position your rating on the scale)

0 1 2 3 4 5 6 7 8 9 10

Extremely unlikely Extremely likely

1. How likely is it that I can inspire teachers in (higher) health care schools to participate in educational Journal Clubs? (please position your rating on the scale)

0 1 2 3 4 5 6 7 8 9 10

Extremely unlikely Extremely likely

1. In my view, the introduction and use of Journal Clubs on topics relating to the qualification of healthcare professions in everyday working life is most likely to fail due to... (please position your rating on the scale)

|  | Least of all | 2 | 3 | 4 | 5 | Most likely |
| --- | --- | --- | --- | --- | --- | --- |
| the willingness of teachers to cooperate | o | o | o | o | o | o |
| supporting the management staff in the vocational schools | o | o | o | o | o | o |
| access to the relevant specialised literature | o | o | o | o | o | o |
| the inadequate English language skills of the teachers | o | o | o | o | o | o |
| someone who regularly organises the Journal Club | o | o | o | o | o | o |
| lack of technical equipment (e. g., for online Journal Clubs) | o | o | o | o | o | o |
| the lack of time in everyday education | o | o | o | o | o | o |

1. The educational Journal Clubs are an important part of the training of educators working in health professions education; they can promote evidence-based (professional) educational action. (please tick as appropriate)

- totally agree
- tend to agree
- rather disagree
- totally disagree

1. I consider participating in a Journal Club on educational or professional issues in the future to be... (please position your rating on the scale)

0 1 2 3 4 5 6 7 8 9 10

Extremely unlikely Extremely likely

1. The fact that I am organising a Journal Club at my future workplace on issues relating to the qualification of healthcare professions is something I consider... (please position your rating on the scale)

0 1 2 3 4 5 6 7 8 9 10

Extremely unlikely Extremely likely

1. What else is important to me regarding Educational Journal Clubs in general... (free text)
